# Observations and Facts Relative to the Practice of Inoculation of the Small-Pox

**Published:** 1786

**Authors:** John Covey

**Affiliations:** Apothecary at Basingstoke, in Hampshire


XII. *Obſervations and Facts relative to the Practice of Inoculation of the Small-pox. Communicated in a Letter to Dr.* Simmons, *F. R. S. by Mr.* John Covey, *Apothecary at Baſingſtoke, in Hampſhire.*

AS you have in the firſt part of the Medical Journal for 1786 communicated ſome obſervations obſervations on the practice of inoculation for the ſmall-pox, I take the liberty of ſending you the following remarks on the ſame ſubject:

I ſhould apprehend little effect could reaſonably be expected from the experiment made by Dr. Houlſton *. Variolous matter, taken on thread or cotton, carefully dried, and kept from the air, will retain its virtue for a conſiderable length of time, but how long I cannot pretend to determine, having never had occaſion to make uſe of any that had been taken more than ſix weeks. That it would retain its virulency for any number of years, is, I think, greatly to be doubted. Matter taken without being dried and cloſely confined from the air, ſoon loſes its efficacy.

The inciſions ſometimes have a partial inflammation for a few days, which then vaniſhes, without producing any illneſs; in this caſe the patient is certainly ſtill liable to infection; but, I believe, it very rarely happens that there is any matter, or even ichor, in the inciſions, in the preſent ſlight manner the ſame are made, without producing the ſmall-pox; provided there was, I ſhould much doubt the poſſibility

* See page 7.

of its communicating the diſtemper to others, by inoculation or otherwiſe. I am convinced that variolous pus, taken on a thread or lancet, or indeed any other extraneous matter, infuſed either by puncture or inciſion into the arm of a patient that has already had the ſmall-pox, will frequently produce inflammation, more or leſs, where inſerted; and ſo will matter that has loſt its virulency, when infuſed into the arm of one who has not yet had the diſtemper. But I have conſtantly remarked, that when the punctured part inflames properly, and is attended with an effloreſcence, rather inclining to a crimſon colour, for ſome diſtance round the ſame, about the eleventh or twelfth day from the inoculation, although the patient ſhould have very little illneſs, and no eruption, yet that he is ſecure from all future infection.

Whether matter taken from a patient in the ſituation deſcribed by Mr. Dawſon is capable, in *general*, of communicating the diſtemper to others, or whether the ſame matter infuſed at a future period into the arm of the patient it was taken from, can produce in him the true ſmall-pox, merits farther inquiry. The caſe recorded by Mr. Dawſon is certainly a ſtrong evidence in favour of the former opinion.

It

It is no uncommon circumſtance, and the ſame has been frequently noticed, for nurſes, or thoſe employed near patients in bad kinds of ſmall-pox, to have a few puſtules, which maturate and turn off in the ſame manner as the real ſmall-pox, notwithſtanding they have already paſſed through this diſtemper, and notwithſtanding the eruption is preceded by no illneſs of any kind; but that matter taken from theſe eruptions will communicate the true diſtemper by inoculation, is a circumſtance, I believe, till lately, not affirmed. I myſelf remember a man, much pitted with the ſmall-pox, who, by his cloſe attendance on his ſon, a fretful boy, under the natural diſtemper, of a bad kind, and, by frequently laying his face cloſe to the child, had an eruption of ſeven or eight large puſtules near one of his eyes, which paſſed through the uſual progreſs, and he was blind with this eye two or three days, but without the attendance of any illneſs whatever. The circumſtance related by Dr. Wright, of the ſix negroes inoculated with matter taken from a puſtule on his own thumb, (he having formerly had the diſtemper) is a convincing proof of the poſſibility of the caſe, provided they had not taken the diſtemper in the natural way, a ſufficient time previous to this ſecond inoculation, to defeat the effect thereof.

Baron Dimſdale has obſerved *, that, by inoculation, the ſmall-pox, taken in the natural way, may be ſuperſeded even ſeveral days after the infection has been taken. This obſervation I have had ſeveral confirmations of; for although the infection very rarely fails of being communicated in the preſent mode of operation, yet this has ſometimes occurred in my practice. In ſuch caſes I have conſtantly, as ſoon as it could be aſcertained, made freſh punctures or inciſions, which have been generally made on the fourth, fifth, or ſixth day, and once in particular ſo late as the ſeventh day; in each of which caſes the patients received the ſmall-pox from the ſecond inoculation, notwithſtanding they were from the firſt liable to receive natural infection from other patients in the ſame houſe, in all ſtages of the diſtemper.

A caſe, ſomewhat ſimilar to the one related by Mr. Mudge †, formerly occurred in my own practice. A young lady, who had a very fa-

* See Vol. II. page 164.

† Diſſertation on the Small-pox, page 80.

vourable number of the ſmall-pox from inoculation, was viſited by ſome friends on the third day from the eruption, and imprudently drank two or three glaſſes of wine with them; in the evening of the ſame day I found her with a high fever, which was followed the enſuing morning by a plentiful crop of the diſtinct ſmall-pox, evidently the production of her own indiſcretion.

To theſe remarks I beg leave to add the following caſe:

Mrs. B. a married gentlewoman, thirty-ſeven years of age, was inoculated, with ſeveral others, on the 29th of March, 1785. The inciſions ſhewing no ſign of inflammation, ſhe was again inoculated with freſh fluid matter the 1ſt of April following: this likewiſe producing no ſign of its having taken effect, the operation was again repeated on the 4th, and ſeemingly with no better ſucceſs; nevertheleſs, on the eighth day, reckoning from the firſt inoculation, ſhe began to complain of head-ach, heat, and the other ſymptoms which uſually precede the eruption of the ſmall-pox. Theſe complaints continued until the 12th, when ſhe had about forty large puſtules, and the illneſs entirely left her. Theſe puſtules maturated kindly, kindly, and dried away on the ſeventh day from the eruption.

From this fact, I think it is evident that the variolous infection may be taken into the circulation from inoculation, although the inciſions may ſhew no ſign of inflammation whatever. That this lady's ſmall-pox aroſe from the firſt inoculation, I believe, will admit of no doubt; for although the places of inſition ſhewed not the leaſt ſign of inflammation from the beginning to the end, yet ſhe ſickened on the ſame day with her companions, and had the diſtemper in the faireſt manner.

I do not remember to have read of a ſimilar inſtance; neither has the like occurred to me in twenty-nine years extenſive practice in this branch of our profeſſion.

If you, Sir, ſhall think theſe obſervations worthy of being communicated to the public, you will do me a favour by inſerting them in the London Medical Journal.

*Baſingſtoke,*

*Hants,*

May 8, 1786.

XIII. *Caſe*

